# Supplementary material for: AVJ16 inhibits lung carcinoma by targeting IGF2BP1
Source: Oncogene. 2025 Jul 8;44(35):3239–54. doi: 10.1038/s41388-025-03449-2 (PMC12375500; doi:10.1038/s41388-025-03449-2)
Supplement: Supplementary file 2 — Supplemental Figures [file 41388_2025_3449_MOESM2_ESM.pdf]

Supplemental Figure 1

Non treated

Rank	Motif	P-value	log P-pvalue	% of Targets	% of Background	STD(Bg STD)
1	UUUCCGAA	1e-85	-1.966e+02	33.45%	27.45%	34.1bp (21.7bp)
2	CUCUCCAGUG	1e-67	-1.544e+02	14.69%	10.90%	34.0bp (22.9bp)
3	UCUUCCAGCAAA	1e-59	-1.369e+02	9.61%	6.71%	33.2bp (20.0bp)
4	UCCUGUUGGA	1e-46	-1.081e+02	4.73%	2.95%	34.2bp (20.9bp)
5	CAGCAGCAAC	1e-46	-1.070e+02	3.11%	1.72%	34.8bp (22.7bp)
6	CUGUAACAGU	1e-45	-1.054e+02	7.53%	5.26%	33.2bp (21.9bp)
7	AUCCCGUACC	1e-45	-1.053e+02	0.25%	0.02%	31.2bp (7.9bp)
8	UGCAUUAGAA	1e-44	-1.022e+02	2.37%	1.20%	34.0bp (18.7bp)
9	GGGAGGCCCUGC	1e-44	-1.014e+02	0.36%	0.05%	30.8bp (17.0bp)
10	CCCUAGGG	1e-38	-8.785e+01	20.87%	17.48%	35.4bp (21.3bp)

Treated

Rank	Motif	P-value	log P-pvalue	% of Targets	% of Background	STD(Bg STD)
1	UUUCCGAA	1e-57	-1.318e+02	23.60%	19.24%	31.7bp (19.8bp)
2	GCCGUUAC	1e-57	-1.314e+02	6.54%	4.21%	34.0bp (21.5bp)
3	CACCGCCG	1e-57	-1.313e+02	12.73%	9.43%	32.8bp (20.7bp)
4	CUGUACAG	1e-51	-1.183e+02	21.70%	17.70%	32.5bp (20.5bp)
5	UUUGCGCA	1e-51	-1.182e+02	6.00%	3.88%	32.3bp (20.5bp)
6	UUUAACCAAA	1e-48	-1.108e+02	8.01%	5.61%	32.0bp (17.5bp)
7	CAUCCAGACACU	1e-44	-1.019e+02	0.20%	0.01%	30.1bp (2.4bp)
8	UCCUCUGG	1e-44	-1.018e+02	6.35%	4.30%	33.9bp (20.0bp)
9	CUUAACGG	1e-43	-1.008e+02	12.48%	9.60%	32.8bp (19.6bp)
10	GGGGCCCC	1e-43	-1.004e+02	15.81%	12.59%	34.0bp (18.3bp)

HOMER analysis of enriched motifs in eCLIP peaks from DMSO (Non-treated) and AVJ16-treated (Treated) H1299 cells.

Supplemental Figure 2

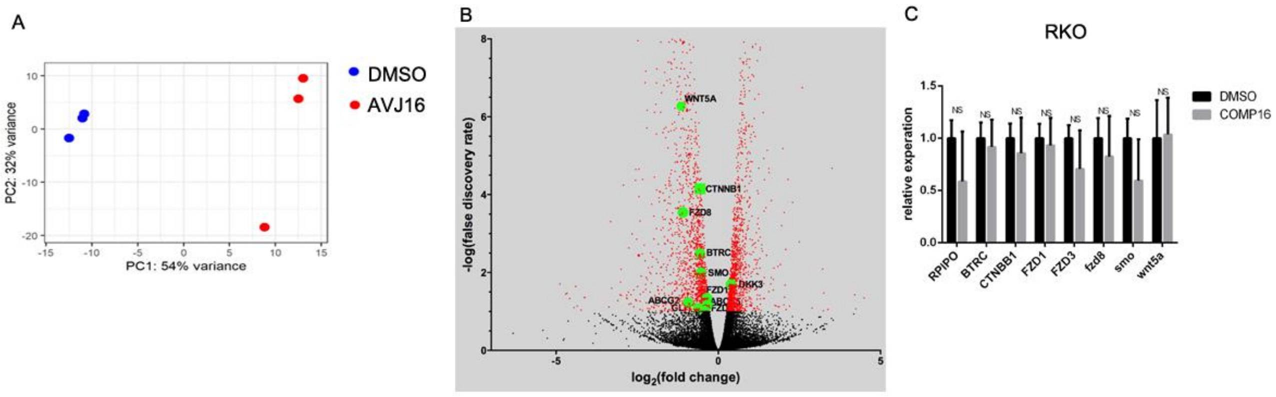

Impact of AVJ16 treatment on the steady state level of RNAs in H1299 cells. RNA-seq was performed on RNA from cells treated with either 1.5μM of AVJ16 or DMSO for 48 hours. (A) Principal component analysis (PCA) plot showing segregation of AVJ16-treated and non-treated cells into two different groups (PC1 54%, PC2 32%). (B) Volcano plot representation of genes after AVJ16 treatment, highlighting some of the known targets of IGF2BP1, including βTrCP1, GLI1, and ABC transporters as well as the RNAs validated by qPCR in Figure 3E. (C) All seven WNT-related genes that were significantly downregulated in H1299 cells upon AVJ16 treatment were not significantly changed in abundance when assayed by qPCR in RKO cells, which do not express IGF2BP1. ns, not significant

Supplemental Figure 3

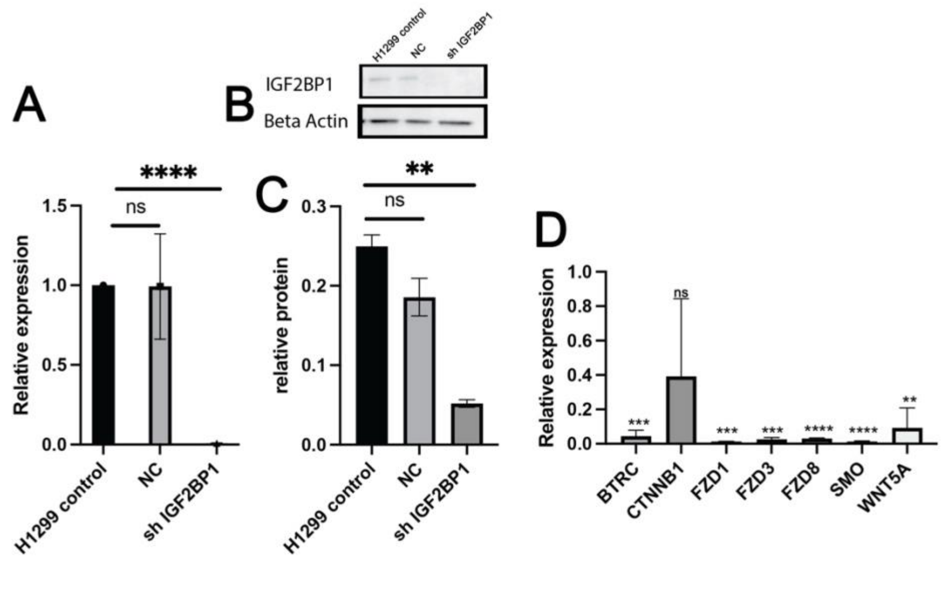

IGF2BP1 knockdown reduces WNT pathway RNA expression levels.

(A) Relative IGF2BP1 mRNA expression in H1299 cells that are non-transduced (*H1299 control*), non-targeting (scrambled) control (*NC*), or IGF2BP1 shRNA-treated (*sh IGF2BP1*), measured by RT-qPCR. (B) Representative Western blot showing IGF2BP1 protein levels following shRNA knockdown, with β-actin as a loading control. (C) Quantification of IGF2BP1 protein levels from Western blot analysis (N = 3). D) Relative expression of key Wnt signaling pathway genes in IGF2BP1 knockdown cells compared to control. **, $p<0.01$; ***, $p<0.001$; ****, $p<0.0001$; ns, not significant.

Supplemental Figure 4

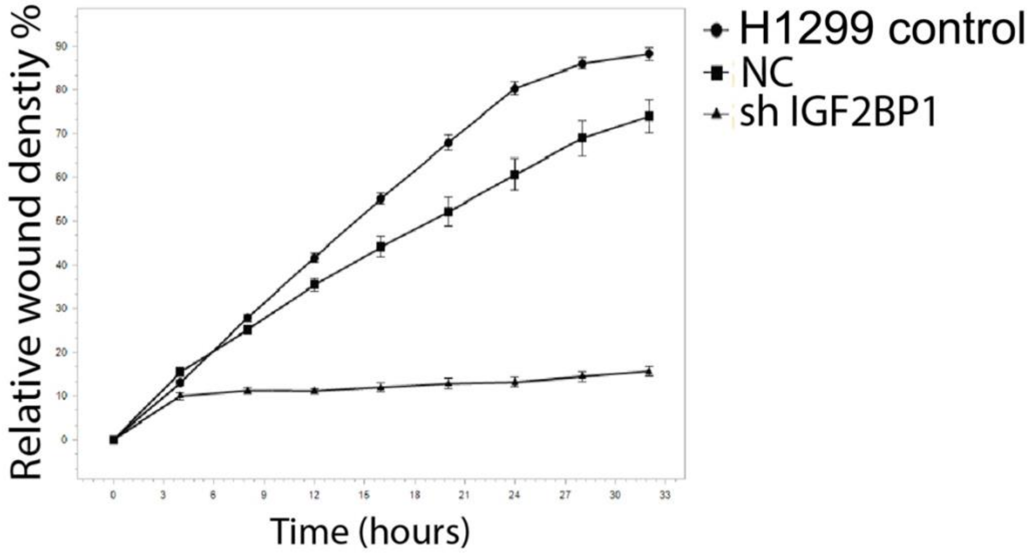

IGF2BP1 knockdown in H1299 cells inhibits wound healing.

Wound healing assay measuring relative wound density (%) over time in non-transduced (*H1299 control*), non-targeting (scrambled) control (*NC*), or IGF2BP1 shRNA-treated (*sh IGF2BP1*). Data are presented as mean ± SEM.

Supplemental Figure 5

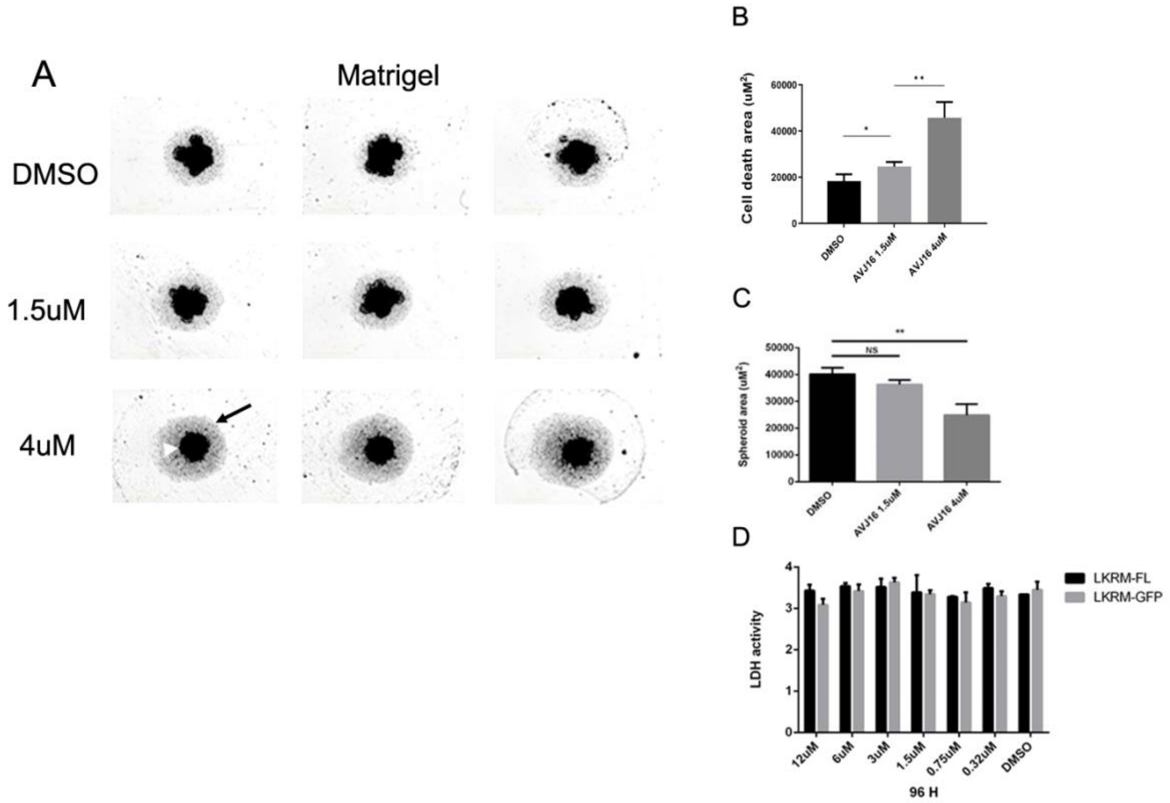

AVJ16 impeded growth and invasion capacity of H1299 cells grown as spheroids in the absence of Matrigel. (A) Spheroids were grown in Matrigel in the presence of DMSO, 1.5μM AVJ16, or 4μM AVJ16. The white arrowhead shows an example of the live cells in a spheroid, and the black arrow points to the dead cells in the same sample. (B) Graph of the area of dead cells surrounding the spheroid as a function of treatment. (C) Graph of the viable cell area as a function of treatment. (D) Toxicity was measured by the presence of LDH in the medium after 96 hours of treatment at the indicated concentrations of AVJ16. *, $p<0.05$; **, $p<0.01$; ns, not significant

Supplemental Figure 6

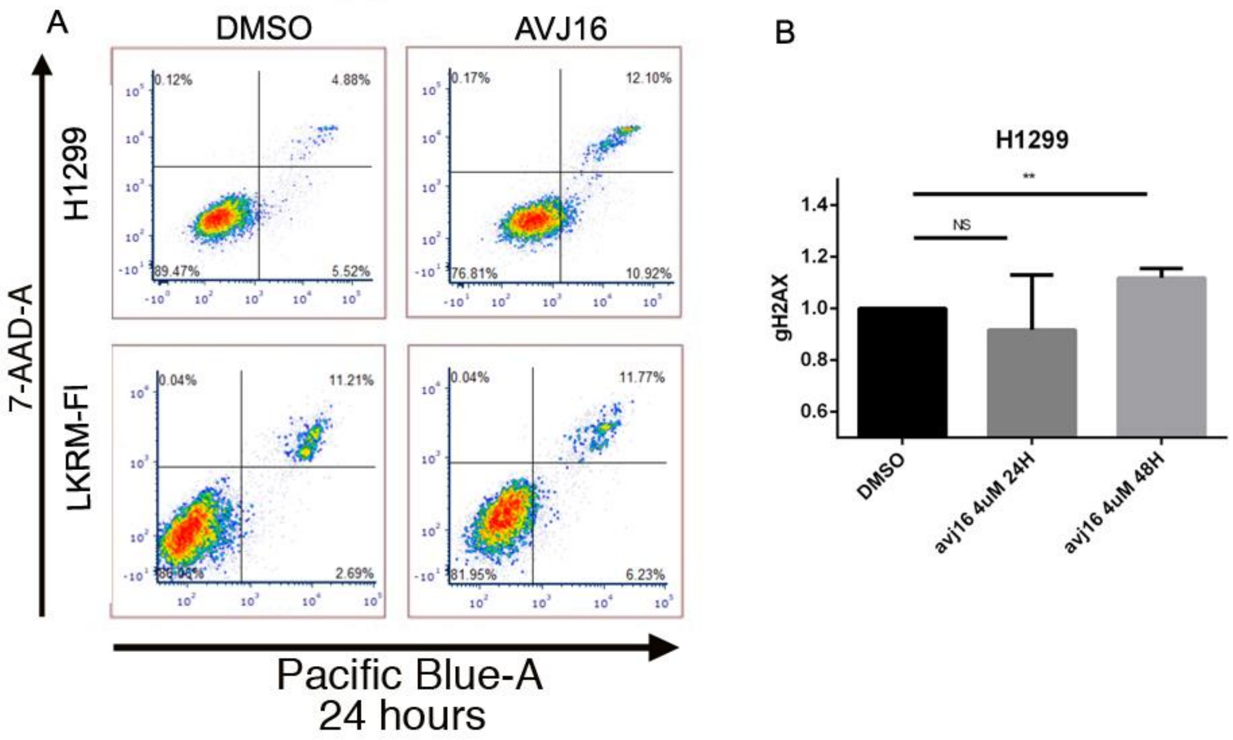

AVJ16 induces apoptosis. (A) FACS sorting was used to detect Annexin/7-AAD-A staining of H1299 and LKR-M-Fl cells treated with DMSO or 4μM AVJ16 for 24 hours. Late apoptotic cells appear in the top right quadrant. (B) H1299 cells treated with 4μM AVJ16 for 24 or 48 hours, and γH2AX levels, indicative of double strand DNA breaks, were measured by western blot analysis and normalized to a DMSO control. *, $p<0.05$; **, $p<0.01$; ns, not significant

Supplemental Figure 7

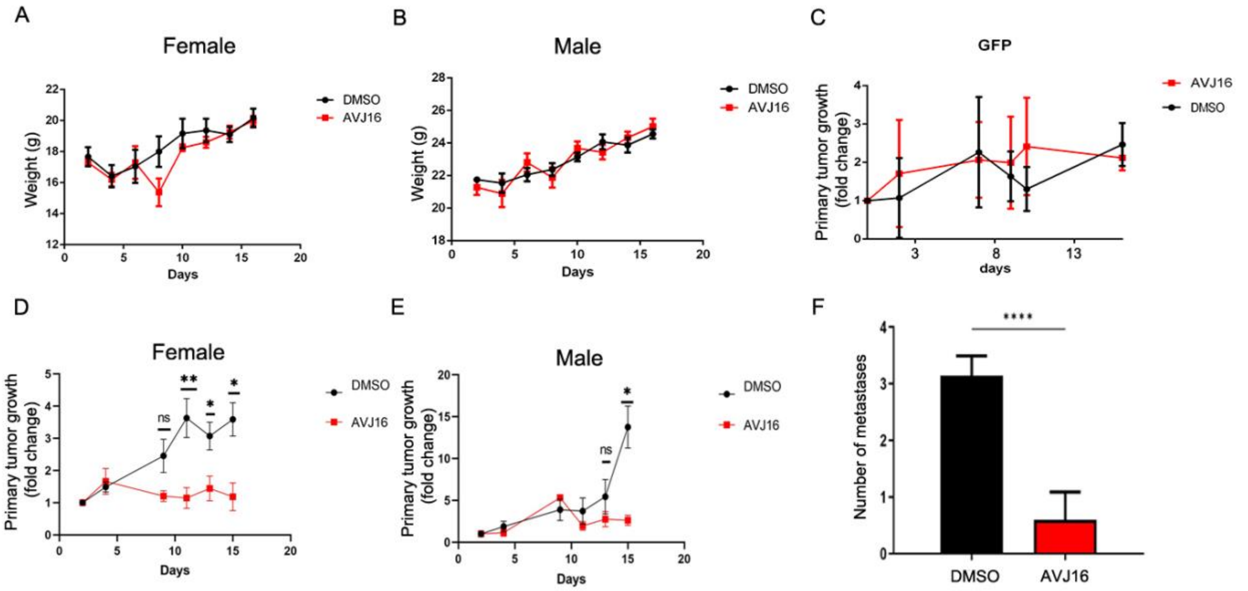

AVJ16 effects on mice xenografts. The female (A) and male (B) mice from the xenograft experiment described in Figure 7 were weighed over the course of the experiment from the point at which AVJ16 injections were begun (from day 12). (C) The effect of IP injections of AVJ16 or DMSO on the growth of LKR-M-GFP xenografts was monitored as in Figure 7. (D-F) Syngeneic mice were subcutaneously implanted with LKR-M-Fl cells and allowed to grow for 12 days, until the site of implantation was visible as a bulge. AVJ16 was injected peritumorally (PT) at the times indicated in Figure 7A. The primary tumor fold increase was measured every two days (time 0 - 12 days after subcutaneous injection of cells) in both females (D) and males (E). (F) The number of lung metastases was counted from lung histology specimens, of mice treated peritumorally (N=3). *, $p<0.05$; **, $p<0.01$; ****, $p<0.0001$; ns, not significant
